# Acellular Urethra Bioscaffold: Decellularization of Whole Urethras for Tissue Engineering Applications

**DOI:** 10.1038/srep41934

**Published:** 2017-02-06

**Authors:** Irina N. Simões, Paulo Vale, Shay Soker, Anthony Atala, Daniel Keller, Rute Noiva, Sandra Carvalho, Conceição Peleteiro, Joaquim M. S. Cabral, Daniel Eberli, Cláudia L. da Silva, Pedro M. Baptista

**Affiliations:** 1 Department of Bioengineering and iBB - Institute for Bioengineering and Biosciences, Instituto Superior Técnico, Universidade de Lisboa, Lisboa, Portugal; 2 Wake Forest Institute for Regenerative Medicine, Winston-Salem, NC USA; 3 Laboratory for Tissue Engineering and Stem Cell Therapy, Department of Urology, University Hospital Zurich, Zurich, Switzerland; 4 Serviço Urologia, Hospital Garcia de Orta, Almada, Portugal; 5 Faculdade de Medicina Veterinária, The Interdisciplinary Centre of Research in Animal Health (CIISA), Universidade de Lisboa, Lisboa, Portugal; 6 Instituto de Investigacion Sanitaria de Aragón (IIS Aragon), Zaragoza, Spain; 7 CIBERehd, Zaragoza, Spain

## Abstract

Patients with stress urinary incontinence mainly suffer from malfunction of the urethra closure mechanism. We established the decellularization of porcine urethras to produce acellular urethra bioscaffolds for future tissue engineering applications, using bioscaffolds or bioscaffold-derived soluble products. Cellular removal was evaluated by H&E, DAPI and DNA quantification. The presence of specific ECM proteins was assessed through immunofluorescence staining and colorimetric assay kits. Human skeletal muscle myoblasts, muscle progenitor cells and adipose-derived stromal vascular fractions were used to evaluate the recellularization of the acellular urethra bioscaffolds. The mechanochemical decellularization system removed ~93% of tissue’s DNA, generally preserving ECM’s components and microarchitecture. Recellularization was achieved, though methodological advances are required regarding cell seeding strategies and functional assessment. Through microdissection and partial digestion, different urethra ECM-derived coating substrates were formulated (*i.e.* containing smooth or skeletal muscle ECM) and used to culture MPCs *in vitro*. The skeletal muscle ECM substrates enhanced fiber formation leading to the expression of the main skeletal muscle-related proteins and genes, as confirmed by immunofluorescence and RT-qPCR. The described methodology produced a urethra bioscaffold that retained vital ECM proteins and was liable to cell repopulation, a crucial first step towards the generation of urethra bioscaffold-based Tissue Engineering products.

Urinary incontinence (UI) affects over 200 million people worldwide and has become more frequent with the increasing age of the population. UI is especially prevalent in women relating to childbirth injuries to muscle, connective tissue and nerves, but also age and obesity, among others^1^. Stress urinary incontinence (SUI) is the most prevalent type (4–35% of women worldwide)^2^,^3^. Most SUI patients suffer from malfunction of the urethra closure mechanism, the so-called external urethral sphincter or rhabdosphincter (RBS)^4^,^5^. The RBS is populated with skeletal muscle myofibers and is located in humans at the mid-urethra (Figure S1a); distinctly, in female pigs, this layer is located at the distal third of the urethra, while in male pigs it is located between the prostatic urethra and the penile urethra^2^,^4^,^5^. The urethra internal mucosal layer and smooth muscle play additional roles on the normal function of the urethra closure mechanism (Figure S1b)^2^,^4^,^5^. Within the scope of this work, only the intrinsic muscle-related causes for SUI were considered relevant.

Current treatments include pharmacological therapies, physiotherapy and surgical techniques with limited efficacy and wide variations between patients while being potentially accompanied by significant side effects (*e.g.* chronic inflammation)^6^,^7^. Hence, alternative therapeutic approaches including tissue engineering using stem/progenitor cells and/or their derived progeny to rescue tissue function are urgently needed. The complexity of urethral functioning and more specifically the skeletal muscle *in vivo* environment^8^ implies therapies that explore the importance of cell-extracellular matrix (ECM) interactions, which are essential for skeletal muscle formation, regeneration and proper function^9^. To do so, naturally-derived and tissue-specific ECM can serve as a basis to develop tissue engineering products for SUI therapies. In 2009, Zhang and colleagues demonstrated the dramatic effect of tissue-specific ECM compounds on the enhancement of cell proliferation and differentiation from each specific tissue^10^. So, such matrices can provide pivotal information about tissue-specific niches and be used to develop a reliable *in vitro* human model of SUI to test new therapies and drugs, since most work has been performed *in vivo,* using animal models^11^. Furthermore, these matrices can serve as a starting material for the development of tissue engineering products.

Naturally derived matrices can be obtained through organ/tissue decellularization to produce bioscaffolds that retain a similar composition, microstructure and biomechanical properties as the native tissue/organ while preserving most of the signaling cues required for organ development, repair and physiological regeneration^12^. These bioscaffolds can be later repopulated with other cell types to generate a ‘neo’ organ^12^. Multiple decellularization techniques have been developed for distinct tissues and organs to find a balance between cell removal and ECM maintenance^13^, because insufficient decellularization holds cellular debris that elicit pro-inflammatory responses. However, on the other hand, aggressive and complete decellularization will bleach growth factors and denature proteins. Perfusion decellularization preserves both biochemical and architectural features of the natural tissue^14^,^15^,^16^. The safety and therapeutic relevance of decellularized bioscaffolds has been demonstrated with the implantation of decellularized bladder and small intestine submucosa ECM^17^,^18^.

Inability to efficiently decellularize urethral tissue^19^ has delayed the generation of an *in vitro* RBS. Hence, the use of acellular native urethra ECM (with preserved RBS) may be vital in the quest of bioengineering physiologically relevant urethral tissues. Ultimately, this will facilitate bioengineering the RBS *in vitro*, something that is rather difficult to accomplish with other synthetic or naturally-derived scaffolds that do not present the same microarchitecture.

Targeting more direct routes into clinical applications, these acellular ECM bioscaffolds have been solubilized to produce muscle specific ECM-derived protein mixtures^20^,^21^. These rich protein mixtures enclose tissue-specific biochemical cues and can be used for clinical product formulation.

Seeded and non-seeded biomaterials have already been used to regenerate urethral tissue while promoting neo-vascularization and stratification of the ingrowing tissue^19^,^22^. In 1998, Kropp and co-workers reported the successful use of small intestine submucosa (SIS) to evoke urethra regeneration in rabbits, focusing on urethral columnar epithelium and smooth muscle bundles^22^. More recently, in 2015, De Filippo and colleagues used acellular collagen matrices derived from decellularized porcine bladders to create seeded tubularized grafts for urethra repair^23^. These structures were seeded with autologous smooth muscle cells and then transplanted into 3 cm urethral defects in rabbits. Although the accomplished results were promising, reporting the formation of new tissue, which is histologically and functionally similar to the native urethra, the study did not focus on the RBS section of the urethra. In fact, regardless the matrix that was used, most reports are mainly focused on smooth muscle and urothelium layers, which are not the key players in the proper functioning of the urethral closure mechanism. To address SUI and take therapies to the next level, it will be crucial to explore the use of skeletal muscle cells (*i.e.* main contributors for sphincter functioning).

To the best of our knowledge, this is the first report of whole urethra decellularization, including the skeletal muscle layer. Inspired by previous work on whole-organ decellularization^14^,^15^,^24^, the main goal of this work was to establish an efficient decellularization method to generate acellular urethra bioscaffolds derived from porcine urethras. In this pilot study, we firstly developed a simple perfusion decellularization method to create urethra bioscaffolds, and secondly assessed cell behavior on (i) the developed bioscaffolds and (ii) bioscaffold-derived soluble products. Thus, the novelty of this study resides on the development of a putative substitute for the use of those scaffolds that do not recapitulate the native tissue but rather use an acellular native-derived bioscaffold, which will virtually hold all the main urethral properties and characteristics. Results show that these bioscaffolds hold the main properties and characteristics of the *in vivo* urethra environment and may serve as a foundation for the development and enhancement of tissue engineering-based SUI therapies.

## Methods

### Study Design

Urethras (n = 17: female, n = 9 and male, n = 8) were obtained from cadaveric female Yorkshire piglets (Group 1: 8.2 ± 3.8 Kg, n = 11) and Duroc male and F1 Large White X Landrace pigs (Group 2: 31 ± 3.9 Kg, n = 6). Cadaveric animals were provided from experimental studies approved by the Animal Care and Use Committee (ACUC) of Wake Forest University Health Sciences in USA and Direcção Geral de Alimentação e Veterinária (DGAV) in Portugal^25^. Animal handling and dissections were performed in accordance with guidelines and regulations of ACUC and DGAV. Thirteen urethras were used for decellularization while four were left intact and used to characterize the native tissue. Porcine urethras were decellularized using a mechanochemical process and subsequently characterized to evaluate the decellularization extent and the composition of the obtained bioscaffold. Envisioning the future generation of urethra bioscaffold-based tissue engineering products, cell behaviour was assessed in two culture platforms: (1) urethra rhabdosphincter bioscaffold sections and (2) urethra rhabdosphincter ECM-derived coatings (i.e. to explore the potential of solubilized ECM for future therapeutic product formulation). The impact of different urethra muscle ECMs (smooth and skeletal) on skeletal muscle fiber formation was further assessed on the ECM-derived coatings experiments to unravel the need of tissue-specific urethra muscle ECMs in further clinical product formulation for RBS-related SUI therapies.

### Decellularization of porcine urethras

A step-wise representation of the decellularization protocol is showed in Table 1. Whole lower urinary tract (LUT) was dissected and prepared for the decellularization apparatus (Supplementary Figure S2): the urethra was cannulated with a 14-gauge (14 G) cannula (Jelco^®^) at its most distal region while a small incision was made at the top of the bladder.

The decellularization apparatus (Figure S2) consisted of a peristaltic pump (Masterflex L/S peristaltic pump with Masterflex L/S easy load pump head and L/S 14 tubing), a magnetic stirrer plate (Fisher-Scientific^TM^,) and a 2 L glass beaker. The system was based on perfusion (40 ml/min) on the inside surface of the tissue and agitation (60 rpm) on the outer surface of the tissue (Supplementary Figure S2). Parameters were set based on previous literature data and preliminary experiments. The LUT was immersed and perfused with distilled water for approximately 30 min. Subsequently, each decellularization solution was tested: 1% Triton X-100 (Sigma©) and 0.1% Ammonium Hydroxide (NH_4_OH, Fischer-Scientific^TM^) (n = 2), 5% Triton X-100 and 0.5% NH_4_OH (n = 2), 0.1% Sodium Dodecyl Sulfate (SDS, Sigma^®^) (n = 2), 0.5% SDS (n = 5) and 1% SDS (n = 2). For 5 days, the organ was perfused with an accumulated volume of 288 L of the tested decellularization solutions that were renewed every 24 h. After the first 48 h, the direction of the perfusion was reversed (Supplementary Figure S2, grey arrows) - cannula moved from the urethra to the incision in the bladder to enhance the decellularization efficiency and homogeneity. The estimated pressure of the perfusate in the urethra was 110.1 N/m^2^, 117.8 N/m^2^ and 113.4 N/m^2^ using distilled water, Triton X-100 and SDS solutions, respectively. The pressure (P) was calculated based on the data shown in Supplementary Table S1. The calculations were made using Equation (1) where F is force (N), Q is flow rate (m^3^/s), V is velocity (m/s) and ρ is the density (Kg/m^3^) of the solution. Distilled water was perfused through the tissue for at least 12 h to wash all the detergent remnants.


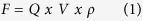


### Urethra Rhabdosphincter Bioscaffold Characterization

Samples of native (n = 4) and decellularized (n = 5, 0.5% SDS and n = 2, 1% SDS) urethra were lyophilized (Labconco^®^) and weighed. DNA was extracted with DNeasy Blood and Tissue Kit (Qiagen Inc.), according to manufacturer’s instructions. DNA concentration was determined using NanoDrop (Thermo Scientific^TM^).

The structure of decellularized bioscaffolds and native urethras was evaluated through scanning electron microscopy (SEM). Briefly, native tissue (n = 2) and decellularized bioscaffold sections (n = 3) were fixed with 4% (v/v) formaldehyde (ThermoFisher©) in phosphate buffered saline (PBS, Gibco^®^) (pH 7.2). Sections were then flash frozen in liquid nitrogen, lyophilized, and observed via SEM (Environmental Scanning Electron Microscope: SEM-Quanta FEG-250, ESEM).

Native (n = 4) and decellularized (n = 5, 0.5% SDS and n = 2, 1% SDS) urethras were fixed with 10% buffered formalin (NBF, Sigma©) and processed for paraffin embedding. Sections (5 μm thick) were stained with hematoxylin and eosin (H&E, Dako^®^). ECM components were identified through immunofluorescence. Sections of native (n = 4) and decellularized (n = 5) urethras were processed for immunofluorescence staining. The antibodies were anti-collagen I-IV (1:50, Southern Biotech), fibronectin (1:200, Santa Cruz Biotech.), elastin (1:50, Santa Cruz Biotech.) and laminin (1:200, Sigma^®^) and secondary Alexa 594-conjugated goat anti-rabbit and donkey anti-goat (1:200, Invitrogen^TM^). Slides were mounted with ProLong^®^ Gold Antifade reagent with DAPI (Invitrogen^TM^). All images were captured on a Leica Microsystems DM4000B upright microscope (Leica Microsystems Inc.) and Image-Pro^®^ Express software (Media Cybernetics Inc.).

Collagen, sulfated glycosaminoglycans (sGAGs) and elastin levels in native (n = 3) and decellularized (n = 3, 0.5% SDS) were quantified using the Sircol Soluble Collagen, the Blyscan sGAG and the Fastin Elastin Assay Kits (Biocolor Ltd), respectively, according to the manufacturer’s instructions. Quantification was determined based on the absorbance at 555 nm, 656 nm and 513 nm, respectively, using a microplate reader SpectraMax M5 Multi-Mode Microplate Reader (Molecular Devices Inc.).

### Recellularization of Urethra Rhabdosphincter Bioscaffolds

Three hundred μm thick cross-sectional acellular urethra bioscaffold sections (n = 25, from 2 urethra RBS bioscaffolds) were produced using a cryostat (C3050S, Leica Microsystems Inc.). Sections had roughly 1 cm^2^ of surface area and were cut from the RBS-containing section of the urethra bioscaffold (*i.e.* containing mucosa and smooth and skeletal muscle ECM). Sections were directly collected into ultra-low attachment Costar^®^ tissue culture plates (Sigma©), washed and sterilized with antibiotics and antimycotics in PBS (1:100, Gibco^®^) and stored at 4 °C in PBS (Gibco^®^). Prior to cell seeding, sections were emulsified in culture medium for at least 30 min at 37 °C. Different types of cells (details in Supplementary Materials and Methods) were individually seeded on the bioscaffolds by deposition with a micropipette. Briefly, sections were first semi-dried to adhere to the tissue culture plate surface. Then. droplets of highly concentrated cell suspensions (around 10 μl each droplet, ~100 μl total with approximately 2 × 10^6^ cells) were deposited on top of each section. The seeded-sections were kept at 37 °C for 30–40 min and then culture medium was carefully added to each tissue culture plate.

Sections were seeded with (2 × 10^6^ cells) human muscle progenitor cells (MPCs, n = 9, from (Zenbio^®^) or freshly isolated from muscle tissue of healthy donors at the University Hospital Zurich (USZ) in Switzerland^26^ (Supplementary Methods) in growth medium. After 2 days, myoblast fusion was promoted using Dulbecco’s modified Eagle’s medium (DMEM) /F12 (Gibco^®^) supplemented with 10% Fetal Bovine Serum (FBS, Gibco^®^). MPCs proliferation was determined using 1:10 WST-1 (Roche©) in growth medium for 4 h at 37 °C (days 3, 5, 7, 9, 11 and 14). Absorbance was measured at 450 nm in a Cytation 3 microplate reader (BioTek^®^). As assay control, an unseeded bioscaffold section was used.

Human bone marrow mesenchymal stem/stromal cells (BM MSC, n = 2, Supplementary Methods^27^) and L929 fibroblasts (n = 2, Leibniz-Institute DSMZ) (cell types similar to the stromal cell populations present in native rhabdosphincter) were seeded separately onto the sections (2 × 10^6^ cells) using DMEM + 10% FBS. Cell proliferation was assessed with anti- Ki-67 FITC (1:200, BD Biosciences).

Stromal vascular fractions (SVF, Supplementary Methods^28^) were cultured (2 × 10^6^ cells) on the sections in the presence of DMEM + 10% FBS (n = 6) or Skeletal Muscle Cell Growth Medium (SkMGM, ZenBio^®^, n = 2) or DMEM + 2% Horse Serum (HS, Gibco^®^, n = 4). All cultures were maintained at 37 °C and 5% CO_2_ with medium changes every 3 days.

### Cell Culture on Urethra Rhabdosphincter Bioscaffold-Derived ECM Coatings

Skeletal and smooth muscle ECM environments (skECM and smECM, respectively) were isolated through microdissection (Supplementary Methods and Supplementary Figure S3). Prior to processing, smECM (n = 4) and skECM (n = 4) were washed overnight with isopropyl alcohol (IPA, Sigma©) followed by distilled water washing. The samples were milled in liquid nitrogen and the ECM was solubilized (10:1) using 4 mg/ml pepsin solution (Sigma©) dissolved in 0.01 M hydrochloric acid (HCl, Sigma©) under constant stirring. After 48 h, pepsin was inactivated by bringing the pH to 8.5–9.0 using sterile-filtered sodium hydroxide (NaOH, Sigma©). The solution was brought to physiological pH by adding sterile filtered hydrochloric acid (HCl, Sigma©) and 10X PBS (Gibco^®^) at 4 °C. smECM and skECM solutions (140 and 180 μg/ml, respectively) were used as coating substrates for MPCs culture. Cells were seeded at 5000 cells/cm^2^ and maintained in growth medium for 48 h. Fiber formation was induced using DMEM/F12 + 10% FBS for 14 days at 37 °C and 5% CO_2_ with medium changes every third day. As controls, MPCs were cultured on culture-grade polystyrene (no coating) or polystyrene surfaces coated with FBS. Cell proliferation was determined using WST-1 (Roche©), as mentioned previously. As assay control, a mixture of growth medium and WST-1 without cells was used.

Cell length and circularity were measured using *ImageJ* 1.49 software from the National Institutes of Health, USA. Measurements were determined based on 6 cells in at least 8 pictures (at least n = 48) *per* condition. Cell circularity was determined according to Equation (2), where Pr is the perimeter and Ar is the area of the cell.


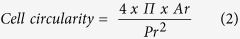


### Immunohistochemistry Staining

Anti-desmin (1:200, Santa Cruz Biotech.), anti-MyoD (1:100, BD Biosciences), anti-Pax3 (1:100, Santa Cruz Biotech.), anti-Myosin Heavy Chain (anti-MHC, 1:200, Biolegend^®^), CD31 (1:30, Dako^®^), anti-Calponin (1:100, Abcam^®^), anti-Vimentin (1:100, Dako^®^) and anti-α-actin (1:180, BD Biosciences) antibodies were used. Novolink Polymer Detection System (Leica Microsystems Inc.) and Vectastain Elite ABC Kit (Vectorlabs^®^) were used for labeling mouse/rabbit and goat antibodies, respectively. Nuclei were counterstained with Mayers’s hematoxylin (Sigma^®^) and images were captured on an Olympus BX51 microscope and a DP21 Digital Camera (Olympus).

### Immunofluorescence Staining

Cultures were fixed in 4% paraformaldehyde (PFA, Gibco^®^) and permeabilized in 0.5% Triton X-100 in PBS. Protein blocking was completed using 5% bovine serum albumin (BSA, Sigma©) in 0.1% Triton X-100. Primary antibodies anti-desmin (1:50, Sigma©), anti-MyoD (1:100, BD Biosciences), anti-α-actinin (1:500, Sigma©) and anti-MyHC (1:1, DSHB) were incubated overnight at 4 °C. Secondary antibody Cy3-conjugated sheep anti-mouse (1:1000, Sigma©) was incubated for 1 h at room temperature in the dark. Cell nuclei were stained with DAPI (1:100, Sigma©). Images were acquired with Leica DM6000 B microscope and LAS AF software both from Leica Microsystems Inc.

### Fiber Formation Assay

Fiber formation (Supplementary Methods) was assessed through Giemsa staining (J.T. Baker^®^). Cultures were fixed in ice cold methanol for 7 min. A 1:20 Giemsa solution was incubated at room temperature for 40–50 min. Images were acquired with Zeiss Axiovert 40 CFL and AxioVision SE64 Rel. 4.9 software. A total of 10 high power fields (HPF) from each condition were analyzed. Results were expressed as fusion rate (%), calculated using Equation (3).


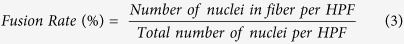


### Statistics

All data was expressed as mean ± standard error of the mean (SEM). One-way ANOVA was used for statistical analysis. Statistical differences between groups from the same experimental set were determined using Turkey post-hoc test. Alternatively, when appropriate, independent t-test or Mann-Whitney U test were used when the samples did not show equal variance in the Levene Test. Statistical analyses were performed using SPSS Statistics software (IBM Corp, Armonk, NY, USA). A p-value < 0.05 was considered significant.

## Results

Harvested urethras had around 5.1 ± 1.1 cm of length in group 1 and 7.1 ± 1.4 cm in group 2. Female urethras had an average length of 4.7 ± 1.6 cm, while male urethras had 6.6 ± 1.8 cm. In this work both female and male porcine urethras were used to test the decellularization method and evaluate its feasibility (only the pelvic section of the male urethra was used – the anatomical localization of the male RBS).

### Decellularization of Porcine Urethras

**Figure 1:**
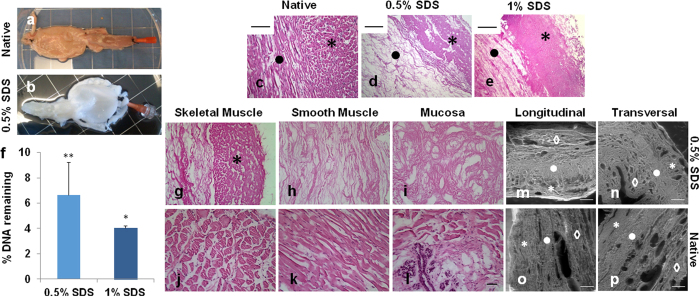
Urethra decellularization. Macroscopic appearance of the lower urinary tract (**a**) before and (**b**) after decellularization. Pictures are from a urethra exposed to 0.5% SDS solution. (**c–e**) H&E staining of (**c**) native and (**d** and **e**) decellularized bioscaffolds (**d**, 0.5% SDS and **e**, 1% SDS). Images are from 5 μm thick sections (*skeletal muscle and ⦁ smooth muscle). Scale bar: 50 μm. (**f**) Quantification of remaining DNA in bioscaffolds using 0.5% and 1% SDS solutions. Results as mean ± SEM, *p ≤ 0.05, **p ≤ 0.01, t-test. Percentage of DNA is referred to the native tissue (assumed as 100% DNA content). Close up of (**g–l**) H&E staining of (**g–i**) native and (**j–l**) decellularized bioscaffolds in the three layers of the urethra: (**g** and **j**, in j marked with*) skeletal muscle, (**h** and **k**) smooth muscle and (**i** and **l**) mucosa. Scale bar: 50 μm. (**m–p**) SEM evaluation of (**m** and **o**) longitudinal and (**n** and **p**) transversal cuts of (**o–p**) native urethra and (**m,n**) 0.5% decellularized bioscaffold (*, skeletal muscle, ⦁, smooth muscle and ♢, mucosa). Scale bar: 250 μm.

Decellularization efficacy was initially determined by macroscopic visualization – tissue became white or transparent due to progressive cell removal (Fig. 1a and b). Only 0.5% and 1% SDS showed macroscopic evidence of decellularization (Supplementary Table S2). Triton X-100 solutions led to a minor discoloration of the tissue and 0.1% SDS solution showed an overall discoloration of the tissue, however, approximately 30% of the organ was not completely white/transparent (data not shown). Tissue decellularization was further confirmed by H&E staining (Fig. 1c–e and Supplementary Figures S4). Tissues subjected to 0.5% and 1% SDS displayed no basophilic purple staining (Fig. 1d and e). Additionally, the absence of relevant DAPI staining (Supplementary Methods and Supplementary Figure S5) confirmed that most cellular nuclear material was removed after emulsification with the detergent. Urethras decellularized with other detergent solutions presented cell remnants by the basophilic dark purple staining (Supplementary Figure S4).

Quantification of cell removal was determined based on DNA quantification in the conditions that showed no cell presence on H&E and DAPI stainings (*i.e.* 0.5% and 1% SDS, Fig. 1f). The 0.5% SDS removed 93 ± 2.6% of total DNA (*i.e.* 629 ± 46.6 ng/mg tissue *versus* 9470 ± 2436 ng/mg tissue in native urethra, p-value = 0.01), while 1% SDS removed 96 ± 0.2% of total DNA (*i.e.* 383 ± 17.4 ng/mg tissue *versus* 9470 ± 2436 ng/mg tissue in native urethra, p-value = 0.02). Since 0.5% SDS has a lower detergent concentration (*i.e.* a presumed milder action over ECM proteins), further studies were pursued using this solution. Detailed evaluation of the decellularization extent in each urethra tissue layer revealed no positive nuclear staining in neither muscle layers (skeletal, Fig. 1g marked with * and smooth, Fig. 1h) nor the mucosa (Fig. 1i) when compared with the native urethra (Fig. 1j–1l). Regardless the group of animals from which the samples were harvested, there were no detectable differences in the decellularization outcome, demonstrating the feasibility of the process in either shorter or longer organs.

### Structure and Biochemical Composition of the Urethra Bioscaffold

**Figure 2:**
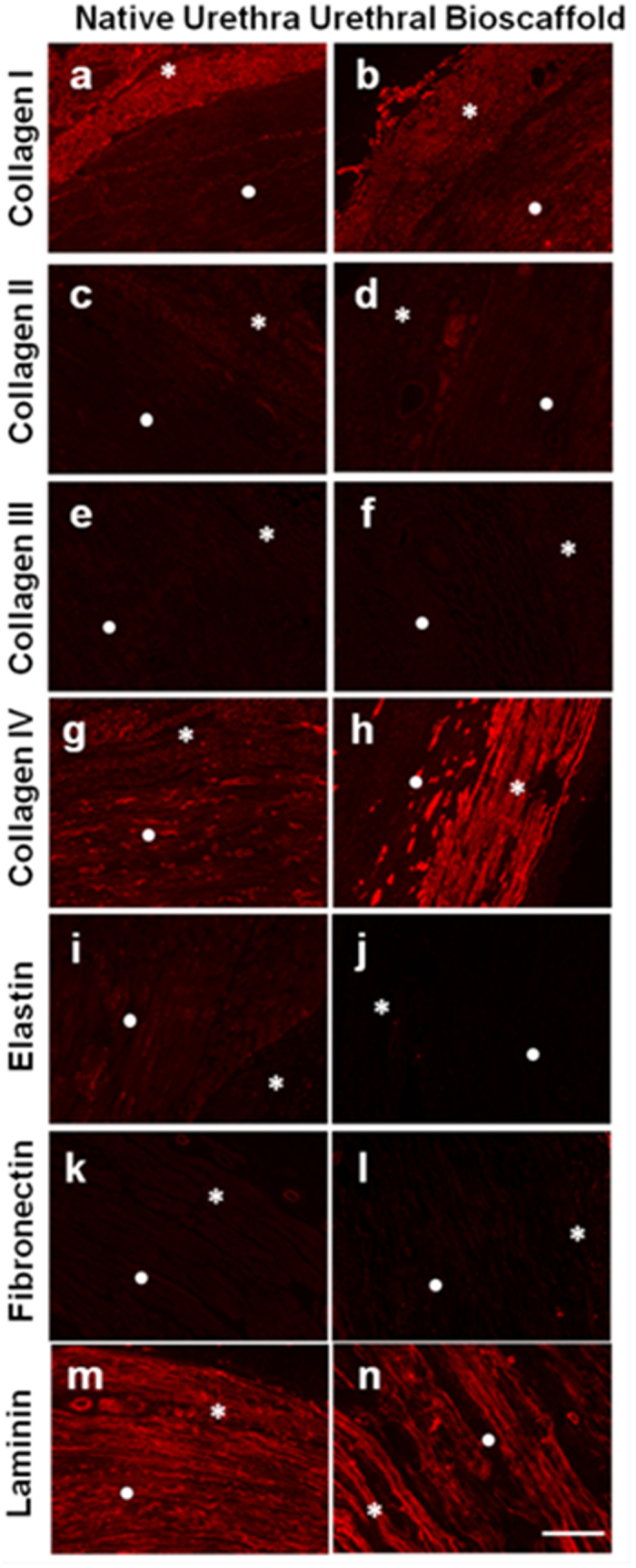
Urethra decellularization. Immunofluorescence staining of (**a**,**c**,**e**,**g**,**I**,**k** and **m**) native urethra and (**b**,**d**,**f**,**h**,**j**,**l** and **n**) bioscaffolds generated using a 0.5% SDS solution (*skeletal and ⦁ smooth muscle layers): collagens I to IV (**a–h**), elastin (**i–j**), fibronectin (**k–l**) and laminin (**m,n**) were tested. Images are from 5 μm thick sections. Scale bar: 100 μm.

SEM analysis revealed a similar ECM microarchitecture between the urethra bioscaffold and the native tissue (Fig. 1m–p and Supplementary Figure S6). Collagen was observed in all areas of the tissue, but mainly in the muscle layers. Collagens I and IV revealed higher abundance in the ECM when compared to the other two collagens that were tested (Fig. 2a,b and g,h *versus* Fig. 2c,d and 2e,f). Elastin and fibronectin showed a lower presence when compared with all the other ECM proteins that were tested (Fig. 2i,j and 2k,l). Laminin distribution was particularly intense in vessels, as well as in both muscle layers (Fig. 2m,n). Overall, there were no major changes when comparing ECM distribution in native urethra and acellular urethra bioscaffolds (Fig. 2 right panel *versus* left panel). Nevertheless, elastin showed an apparent decrease in the bioscaffold (Fig. 2i and j). ECM protein quantification (Table 2) indicated that 20.8 ± 2.45% of the dry weight of the urethra bioscaffold was collagen, which was significantly higher (p-value = 0.01) than the quantity found in native tissue (5.0 ± 0.65%). The apparent increase is explained by the efficient removal of cellular material in the decellularization process, changing the final ratio of ECM/cellular proteins. Similarly, GAGs composed 5.4 ± 0.35% of the urethra bioscaffold while only 2.8 ± 1.22% (p-value = 0.03) were detected in the native urethra. Finally, elastin quantification confirmed a significant decrease (p-value = 0.01) of this protein in the bioscaffold treated with 0.5% SDS (18.3 ± 1.63%) when compared to the native tissue (37.7 ± 5.15%).

### Recellularization of the Urethra RBS Bioscaffolds

Regardless the final application, bioscaffold recellularization is unequivocally required. In this pilot study, recellularization was mainly evaluated through cell adhesion and behavior of MPCs, BM MSC and L929 cells on the urethra bioscaffolds sections. The initial cell seeding density in the bioscaffolds was determined based on previous knowledge and other work reported in the literature^29^,^30^,^31^. Fiber fusion potential of MPCs used to repopulate the bioscaffolds was previously tested in *in vitro* standard culture conditions (methods described in Supplementary Methods). In standard culture conditions, MPCs fused and formed multinucleated fibers that expressed the main skeletal muscle markers (Supplementary Figures S7 and S8) and spontaneously contracted after 14 days of myogenic induction (Supplementary Video S1).

**Figure 3:**
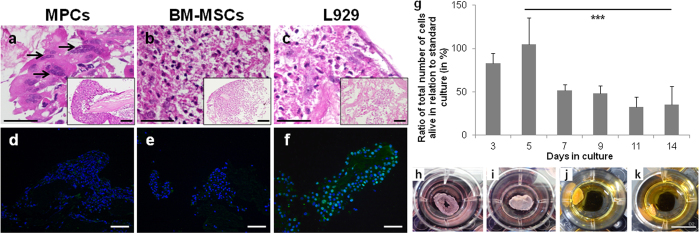
Recellularization of urethra bioscaffolds. (**a–c**) H&E staining and (**d–f**) Ki-67 (green) immunofluorescence staining of recellularized bioscaffolds using (**a** and **d**) MPCs, (**b** and **e**) BM MSCs and (**c** and **f**) L929 fibroblasts (14 days in culture, black arrows indicate multinucleated fibers). Scale bar: 100 μm. Nuclei were stained with DAPI (in blue). (**g**) Ratio of total cells alive in relation to MPCs in standard culture conditions (WST-1 assay, at 450 nm). Results as mean ± SEM (n = 3), ***p ≤ 0.001, ANOVA. (**h–k**) Morphological alterations of the bioscaffold due to contraction along time culture (**h**, day 1, **i**, day 3, **j**, day 7 and **k**, day 14). Scale bar: 1 cm.

MPCs were able to repopulate the bioscaffold sections and differentiate into multinucleated fibers (Fig. 3a, black arrows), especially in the peripheral/external surface of the bioscaffolds, where the RBS is located. BM MSC and L929 cells showed a more homogenous pattern throughout the bioscaffold (Fig. 3b and c). Cells did not only repopulate the surface area of the bioscaffold but also penetrated the 3D structure as confirmed by the H&E staining of sequential recellularized bioscaffold sections (data not shown). Cellular expansion in the bioscaffold was determined via immunofluorescence staining for the proliferation marker Ki-67 (Fig. 3d–f). This showed L929 cells proliferating in the scaffold (Fig. 3f), even after 2 weeks in culture. The seeded MPCs and BM MSC showed no positive staining for Ki-67 after 2 weeks in culture (Fig. 3d and e). For MPCs, the lack of cellular proliferation at day 14 is in accordance with the observed cellular differentiation/myofiber formation (Fig. 3a).

Cell proliferation in the bioscaffolds was assessed using MPCs (Fig. 3g). Maximum cell proliferation (around the values attained in standard culture conditions) was reached after 5 days (Fig. 3g), while after this point, cell proliferation significantly decrease around 50% (p-value = 0.001). This may be related to bioscaffold contraction and “collapse” due to initial cell proliferation. At day 3 (Fig. 3i) the bioscaffold already showed contraction and decreased surface area when compared to day 1 (Fig. 3h). Bioscaffold contraction and “collapse” was even more evident after day 7 (Fig. 3j and k). Nevertheless, since the 3D cultures were performed under static conditions, diffusional limitations might have occurred turning nutrient/oxygen supply to cells and metabolic waste removal inefficient.

**Figure 4:**
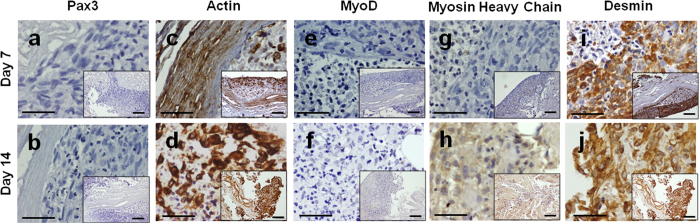
Recellularization of urethra bioscaffold. Immunohistochemistry of bioscaffolds recellularized with MPCs after 14 days of myogenic induction. The presence of (**a** and **b**) Pax3, (**c** and **d**) α-actin, (**e** and **f**) MyoD, (**g** and **h**) MyHC and (**i** and **j**) desmin was evaluated at days 7 and 14. Scale bar: 100 μm.

Myoblasts that repopulated the bioscaffold fused to form multinucleated fibers, expressing the main skeletal muscle markers after 2 weeks of myogenic induction (Fig. 4). Fusion rate was 19 ± 3% in the bioscaffolds, showing 6.8 ± 0.8 fibers/HPF and 3.6 ± 0.1 nuclei/fiber. These values were not significantly different from the ones attained in standard culture conditions (p-value > 0.05, 34 ± 3% fiber fusion, 3.9 ± 0.5 fibers/HPF and 7.8 ± 1.2 nuclei/fiber).

The behavior of heterogeneous cell populations in the bioscaffolds was assessed with adipose-derived SVF populations (Supplementary Figure S9). In the presence of standard culture medium (DMEM + 10% FBS), these cells gave rise to structures that were positive for the presence of actin, calponin, MyoD, MyHC (although the staining is weak) and vimentin (Supplementary Figure S9a–S9d and S9f). Importantly, SVF populations gave rise to CD31 + structures, which are crucial for tissue formation and regeneration. After myogenic induction (DMEM + 2% HS), SVF-derived cells gave rise to structures positive for both muscle (actin, calponin, MyoD and MyHC) and stromal (vimentin) markers (Supplementary Figure S9g–S9k).

### *In Vitro* Culture using Urethra ECM-based Coatings

To explore further urethra bioscaffold applications, we used protein extracts from both urethra skeletal (skECM) and smooth (smECM) muscle ECM as coating substrates for *in vitro* culture. By separating the two tissue-specific urethra muscle ECMs, we assessed the impact of each biochemical environment on skeletal muscle fiber formation and protein expression.

**Figure 5:**
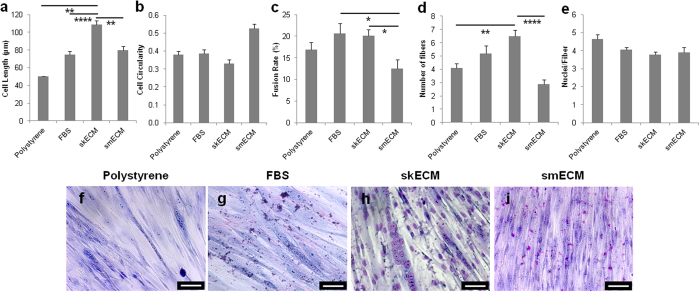
Evaluation of cell morphology and fiber formation on urethra ECM coatings. (**a**) Cell length (μm) and (**b**) circularity were determined for a total of 60 cells *per* condition. (**c**) Fiber formation (n = 10 HPF) was evaluated through fusion rate (%), (**d**) number of fibers and (**e**) number of nuclei *per* fiber. Results as mean ± SEM, *p ≤ 0.05, **p ≤ 0.01, ****p ≤ 0.0001, all others are not significant, p > 0.05, ANOVA. (**f–i**) Giemsa stainings were used to quantify fiber formation. Scale bar: 50 μm.

MPCs remained viable in all ECM substrates (Supplementary Methods and Supplementary Figure S10), showing no significant differences in cell proliferation (p-value > 0.05, Supplementary Figure S10). Microscopic visualization indicated apparent cell morphology differences upon initial cell seeding on the different substrates (Supplementary Figure S10). MPCs showed a significant longer morphology on skECM coatings (109 ± 4.9 μm) when compared to all other coatings (50 ± 3.1 μm, p-value = 0.0001 and 80 ± 4.3 μm, p-value = 0.003 for FBS and smECM, respectively, Fig. 5a), while showing no significant differences in cell circularity (Fig. 5b). Still, MPCs on smECM coatings were significantly more circular when compared to all other coatings (0.53 ± 0.03, p-value = 0.001, Fig. 5b).

The highest fiber fusion rates were obtained in skECM (20 ± 1.4%) and FBS (21 ± 2.4%) substrates (Fig. 5c). The fusion rate on skECM coatings was not significantly different from the control conditions (p-value = 0.074). Still, the fusion rate acquired using skECM was significantly higher than the one achieved using smECM substrates (p-value = 0.035, Fig. 5c). Additionally, the number of fibers present in skECM coatings was significantly higher than in smECM (6.5 ± 0.52 *versus* 2.9 ± 0.31, p-value = 0.0001, Fig. 5d) and non-coated polystyrene (4.1 ± 0.34, p-value = 0.008, Fig. 5d). There were no differences in the number of nuclei per fiber (p-value = 0.059, Fig. 5e). All calculations were based on image analysis of Giemsa stainings (Fig. 5f–i).

**Figure 6:**
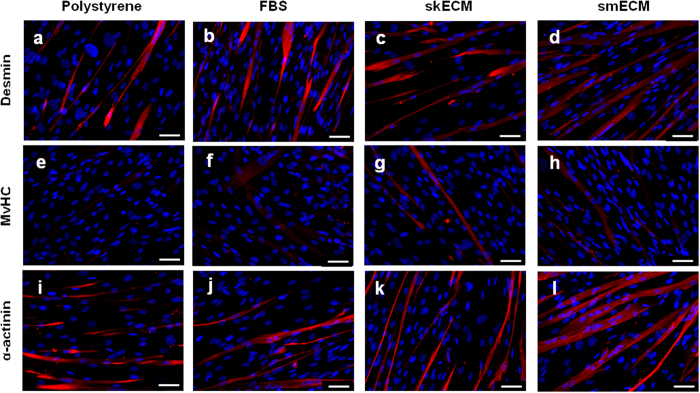
Expression of skeletal muscle-related markers in MPCs cultured on urethra ECM coatings. (**a–d**) Desmin, (**e–h**) MyHC and (**i–l**) α-actinin (red) was assessed through immunofluorescent staining on MPCs in differentiation for 14 days. Nuclei were stained with DAPI (blue). Scale bar: 50 μm.

The expression of skeletal muscle-related proteins and genes was evaluated in all substrates (Fig. 6 and Supplementary Figure S11, Supplementary Methods for RT-qPCR). While at the gene level no major differences were observed (Supplementary Figure S10), the immunofluorescence staining revealed differences according to the type of substrate (Fig. 6). The presence of desmin, MyHC and actinin was confirmed in all cultures. Still, in all conditions MyHC showed an apparent lower expression when compared to desmin and actinin (Fig. 6e–h *versus* all others). The stainings revealed an apparent higher expression of all proteins in MPCs cultured in urethra ECM (either skECM or smECM) when compared to the control conditions (Fig. 6). This was particularly evident for MyHC (Fig. 6g–h *versus* Fig. 6e–f). All together, these preliminary data indicates that urethra-specific ECM has an impact on cell behavior in culture, in a way that MPCs show better performance in ECM environments derived from actual skeletal muscle ECM.

## Discussion

The biochemical, architectural and physiological complexity of the urethra environment, in which normal functioning is highly dependent on cell-ECM interactions^32^,^33^,^34^, requires tissue engineering strategies that mimic, as much as possible, the native environment of the organ.

In recent years, tissue engineering has successfully progressed into the use of decellularized matrices^12^,^13^,^35^,^36^. Such matrices retain chemical and biological cues, while showing similar composition and microstructure as the native tissue^13^,^37^. These properties are crucial for tissue engineering approaches, making the decellularized matrices a more physiological environment for the target cells^13^. The decellularization protocol and detergent are not universal and have to be optimized for each organ/tissue and individual applications/goals due to intrinsic factors such as cellular density, specific density, lipid content and thickness^38^.

To the best of our knowledge, this is the first report of whole urethra decellularization, including the RBS layer. Several groups have performed skeletal muscle decellularization of tissue fragments^21^,^39^,^40^. We used porcine urethras that are an excellent animal model to study and evaluate treatments for sphincter deficiencies due to its functional similarities with the human urethra^4^,^5^. We present a dynamic decellularization scheme for porcine urethras based on agitation and perfusion (of the external and internal surfaces of the organ, respectively). Our system combines perfusion forces and chemical emulsification in order to promote a more efficient and homogeneous cellular removal from the thick urethra fiber mesh^13^,^38^. Previous work on vascular tissue has demonstrated that the decellularization of thick and dense tissues, based exclusively on agitation is very limited and ineffective on generating a completely acellular bioscaffold^41^. This is because thicker tissues tend to decellularize well at the surface, while the cells at the inner core are preserved (the decellularized matrix at the surface forms a resistant layer preventing efficient access to the deeper tissue parenchyma). Additionally, many describe that perfusion forces contribute to a higher penetration of the chemical agent into the parenchyma increasing cell removal^19^,^38^,^41^. As Consolo and co-workers reported, mechanochemical decellularization processes are more effective on removing cellular material and maintaining the main tissue characteristics and properties than chemical approaches alone^42^. The male spongy urethra has been previously decellularized by Feng and colleagues using a 1% Triton X-100 solution (with no report on cell removal efficiency)^43^, still there are no reports on the decellularization of the urinary sphincter. In fact, most of the reports on skeletal muscle decellularization are not focused on urethra-derived skeletal muscle, but rather on abdominal and leg muscles^39^,^44^.

Based on the work of several groups on the decellularization of other organs/tissues^14^,^24^,^45^ and with the aim of keeping the decellularization process as simple as possible, we used two detergents: Triton X-100 and SDS. Triton X-100 disrupts lipid-lipid and lipid-protein bonds, having a milder action over the bonds between ECM proteins while SDS acts over protein-protein bonds, and so has a harsher effect on the ECM^13^,^38^. In this work, Triton X-100 was not effective on generating acellular bioscaffolds. Consequently, the lower concentration of SDS that actually removed most of the cellular material from the tissue (*i.e.* 0.5% SDS) became the solution of choice. The use of this detergent in combination with two mechanical forces resulted in the removal of more than 90% of total DNA, generating acellular urethra bioscaffolds with no visible cellular content on H&E and DAPI stainings. These results are similar to others reported in the literature concerning liver, heart and lungs decellularization^14^,^24^,^45^. Recently, it was proposed the three-part criteria for tissue decellularization: (1) no visible nuclei in H&E and DAPI staining, (2) no more than 50 ng DNA/mg dry weight and (3) the remaining DNA should not exceed 200 base pair in length^38^. Though, these criteria are still debatable and not followed by all groups and published reports^46^. Considering further clinical applications and according to the future criteria that will be set for decellularized tissues, the protocol developed here will need to be further explored and optimized. Microstructure analysis showed that the decellularization method yielded an apparently intact and unaltered ECM microarchitecture in the bioscaffold when compared to the native urethral tissue. Immunofluorescence confirmed no major qualitative loss of ECM components, although elastin showed ~50% significant loss in the bioscaffold. Elastin is responsible for mechanical strength and elasticity, essential properties for the normal contractile function of the urethra closure mechanism^47^ so it can be anticipated that this loss may have an impact on the mechanical properties of the bioscaffold (not assessed). Nevertheless, as this is majorly a structural protein, presumably it may not compromise recellularization. Furthermore, seeded cells will have the ability to remodel the bioscaffold by secreting matrix metalloproteinases (MMPs) and *de novo* ECM molecules, potentially restoring the native composition of urethra RBS bioscaffold^29^. Still, elastin expression is reduced with urogenital tissue aging^48^, so the chances of elastin restoration by the patient’s autologous cells *in vitro* is currently unknown. Collagens I and IV have been described as the most abundant components in urethra’s ECM as these provide structure and support to cells^20^. In this particular study, there was no evident loss of collagens. Fibronectin encodes cell adhesion peptide motifs (*e.g.* RGD motifs that bind to integrins)^13^,^49^ and it is especially important not only for cell adhesion, but also for cell growth, migration and differentiation. This protein is insoluble in most decellularization solutions and it is preserved in most decellularized organ scaffolds^49^, as it seems to be the case in the decellularized urethra RBS bioscaffolds described here. Finally, laminin is also vital for cell adhesion due to YIGSR and IKVAV polypeptide sequences that are similarly involved in cell adhesion^13^. Thus, the maintenance of these proteins is thought to be crucial to facilitate recellularization, by inducing cell adhesion, proliferation and migration^13^. Here, we only tested a strict group of ECM proteins, still it is important to address that there are virtually hundreds, if not thousands, of proteins in these naturally-derived ECM bioscaffolds^50^.

In this pilot study, SDS remnants were not quantified. Still, it has been shown by others that after prolonged washing the residual SDS content of a 1% SDS decellularized tissue is nearly negligible^51^. Additionally, the presence of porcine viruses such as porcine endogenous retroviruses (PERV), hepatitis E and circoviruses was not assessed. Still, recent reports have shown that the decellularization of porcine livers using 0.1% SDS eliminates swine leukocyte antigens as well as PERV DNA^52^. Furthermore, the required doses of gamma-irradiation (1.5–3.5 Mrad) enforced by most regulatory agencies (FDA and EMA) in the sterilization of naturally-derived matrices for human application are able to completely eliminate all the infectious agents from these products^53^,^54^. Ultimately, the decellularization protocol developed here can potentially be further adjusted and optimized for the decellularization of human urethras, yielding a xenogeneic-free product for clinical settings.

Together, the decellularization data shows that the simple mechanochemical decellularization method proposed here created acellular urethra bioscaffolds that in general retained the overall composition of the native tissue.

The recellularization potential of the urethra bioscaffolds and cell behavior upon culture were assessed. MPCs, BM MSCs, and SVF were seeded on the bioscaffolds due to their important role in muscle and sphincter formation as well as tissue regeneration^55^,^56^. These preliminary recellularization studies revealed that the bioscaffolds provided a suitable environment for cell adhesion, proliferation, and survival confirmed by the positive H&E and Ki-67 stainings and WST-1 assay. The presence and maintenance of these cells for up to 2 weeks in the bioscaffolds demonstrated that the decellularization process potentially maintained the primary biochemical cues of the native tissue, allowing cells to repopulate the urethra ECM. It is noteworthy that the side-by-side use of immortalized cell lines (*i.e.* L929 fibroblasts) and primary cell cultures (*i.e.* all others) may introduce discrepancies in the results, since immortalized cell lines display an abnormal behavior, representing only surrogate models for primary cells^57^. MPCs were able to proliferate and survive in the bioscaffold for up to 14 days, despite the notorious contraction and collapse of the 3D sections. This may be explained by cell contraction forces that were created after the formation of strong cell-cell and cell-ECM networks during proliferation^58^. Ultimately, the collapse and the formation of an “ECM mass” may have led to inefficient nutrient supply and waste removal, which may explain the lower proliferation levels achieved after day 5. Nevertheless, the MPCs were able to fuse and form multinucleated structures expressing skeletal muscle markers, indicating that these cells were able to differentiate in the bioscaffold, showing lower proliferation rates (due to differentiation). When compared to standard culture conditions, fiber fusion in the bioscaffolds was lower (around 50% less). Still, the non-homogeneous distribution of cells and the existence of areas that are not conducive to the differentiation of skeletal muscle may explain the lower levels. Additionally, the 3D configuration of the bioscaffolds turns accurate quantification of fiber formation more difficult when compared to standard culture conditions where fibers are clearly seen and the nuclei inside each fiber can be easily identified.

Further seeding experiments using other cell types of interest (*i.e.* SVF) reassured the potential of the acellular urethra bioscaffolds to support tissue formation. The rationale of using a heterogeneous cell population was not only due to its attractiveness for tissue engineering applications (*i.e.* rich content in stem and progenitor cells) but also to assess differential cell deposition in the bioscaffold. Through immunohistochemistry, it was confirmed that the SVF population gave rise to stromal, muscle and CD31-positive structures in the bioscaffold mesh. Noteworthy, the presence of endothelial-like structures was only detected in specific areas of the bioscaffold, indicating that in fact cells may be more attracted to some areas of the ECM mesh than others.

Together, the recellularization data indicates that the decellularization process produced an ECM matrix that retained the complex biochemical and 3D architecture of the native urethra, providing a suitable environment for cell proliferation and differentiation. This matrix provided a microenvironmental blueprint for the seeded cells, shaping and influencing their behavior in the bioscaffold.

In the present study, the use of 3D bioscaffold sections was justified in order to simplify the studies of cell adhesion, proliferation and fiber formation. Nevertheless, the future goal would be the recellularization of whole urethra bioscaffolds using tailor designed bioreactors to create an *in vitro* urethra model.

The precise microdissection of the RBS from other urethra segments allowed the study of the importance of muscle tissue-specific urethra ECM on skeletal muscle cell behavior and differentiation. After solubilization, ECMs were used as coating substrates in standard culture conditions. MPCs showed no differences in cell proliferation and viability when cultured in skECM or smECM (or even the control conditions). Importantly, upon cell seeding, cells showed different phenotypes according to the substrate in which they were cultured. Cells cultured on skECM were significantly longer than all others while the ones on smECM were more circular. The fiber formation assay revealed a significant 8% higher fusion rate on skECM than on smECM as well as higher number of fibers that revealed positive staining for the main protein skeletal muscle markers (not so evident at the gene level), demonstrating that the skECM protein mixture provided a better support for cell spreading, alignment and fiber formation. Together, the preliminary data using smECM and skECM coatings demonstrates that, as reported in other tissues^10^, the use of tissue-specific ECM compounds is crucial to enhance the formation and regeneration of each specific tissue. Thus, if the target would be a RBS-related SUI therapy, the focus will need to be urethra skECM, besides whole urethra ECM, in order to potentiate the formation of a functional RBS.

Overall, despite the need for improved bioengineering strategies that potentiate myogenic induction (*e.g.* using adhesion proteins and/or specific signaling molecules to potentiate myogenesis), the results presented here are a first solid step in that direction. The acellular bioscaffold developed in this study serves as a starting platform for further studies on urethra/RBS bioengineering since it maintains the major characteristics of the native tissue. Ultimately, these bioscaffolds may be used for the development of RBS-ECM based products for tissue engineering strategies to treat SUI in order to potentiate skeletal muscle formation or to be directly used to develop urethra/RBS models *in vitro*.

Considering the importance of innervation in musculoskeletal regeneration and function^59^, it will be crucial to assess the behavior of nerve cells and neo-innervation in the urethra bioscaffolds developed here. To improve scaffold’s recellularization, surgical urethra dissection along with its vasculature will have to be explored as it has been with other organs/tissues^5^,^14^,^38^,^60^. The use of vasculature for recellularization would improve cell penetration into deeper regions of the urethra rhabdosphincter bioscaffold.

## Conclusion

This pilot study provides a simple mechanochemical method to decellularize porcine urethras (containing the urethral rhabdosphincter - RBS), which yielded an acellular bioscaffold that retained the microstructure and overall biochemical composition of the native tissue. Despite the need for further improvements on recellularization, the produced bioscaffold was suitable for cell adhesion, proliferation and fiber formation yielding human skeletal muscle-like structures expressing the typical myogenic markers. Cell culture on urethra smooth muscle and skeletal muscle derived substrates evidenced that tissue-specific ECM enhances cell behavior and ultimately cell function.

Finally, these bioscaffolds will serve as ideal platforms to extend the experience regarding cell seeding strategies, ECM composition adjustment, function rescue and alternative cell-based therapies.

## Additional Information

**How to cite this article**: Simões, I. N. *et al*. Acellular Urethra Bioscaffold: Decellularization of Whole Urethras for Tissue Engineering Applications. *Sci. Rep.* **7**, 41934; doi: 10.1038/srep41934 (2017).

**Publisher's note:** Springer Nature remains neutral with regard to jurisdictional claims in published maps and institutional affiliations.

## Supplementary Material

Supplementary Information

Supplementary Video V1

## Figures and Tables

**Table 1:** Step-wise representation of the established protocol for the decellularization of porcine urethras.

Step	Time (h)	Conditions	Aim
Urethra dissection	0.5	N/A	Remove adipose tissue
Urethra cannulation	0.2	14 G cannula and surgical suture	Fix the cannula to one extremity of the urethra
Mount decellularization apparatus	0.2	Supplementary Figure S2	Attach the cannula to the perfusion tubing system
Wash urethra	0.5	Distilled water: 40 ml/min + 60 rpm	Wash out blood and debris
Add detergent solution	0.1	Detergent solution (details in Methods)	Replace distilled water by detergent solution
Start decellularization process	48	40 ml/min + 60 rpm	Remove cellular components of the tissue and preserve the ECM
Change perfusion direction	0.2	Change the cannula from one extremity of the urethra to the other	Allow both extremities to be equally exposed to the perfusion forces
Continue decellularization process	72	40 ml/min + 60 rpm	Remove cellular components of the tissue and preserve the ECM
Wash bioscaffold	≥ 12	Distilled water: 40 ml/min + 60 rpm	Remove detergent remnants
Total	≥ 133.7		

N/A: not applicable.

**Table 2:** Quantification of ECM proteins through colorimetric assays in native urethra (n = 3) and urethra bioscaffolds (n = 3).

	Native Urethra	Urethra Bioscaffold
Collagen (%)	5.0 ± 0.85	20.8 ± 2.45**
GAGs (%)	2.8 ± 1.22	5.4 ± 0.35*
Elastin (%)	37.7 ± 5.15	18.3 ± 1.63**

Results as mean % in total dry mass ± SEM, ^*^p ≤ 0.05, ^**^p ≤ 0.01, Mann-Whitney U test.
