# Supplementary material for: Acellular Urethra Bioscaffold: Decellularization of Whole Urethras for Tissue Engineering Applications
Source: Sci Rep. 2017 Feb 6;7:41934. doi: 10.1038/srep41934 (PMC5292742; doi:10.1038/srep41934)
Supplement: Supplementary Information [file srep41934-s1.pdf]

Acellular Urethra Bioscaffold: Decellularization of Whole Urethras for Tissue Engineering Applications

IRINA N. SIMÕES[a,b,c], PAULO VALE[d], SHAY SOKER[b], ANTHONY ATALA[b], DANIEL KELLER[c], RUTE NOIVA[e], SANDRA CARVALHO[e], CONCEIÇÃO PELETEIRO[e], JOAQUIM M. S. CABRAL[a], DANIEL EBERLI[c], CLÁUDIA L. DA SILVA[a] AND *PEDRO M. BAPTISTA[b,f,g]

[a]Department of Bioengineering and IBB - Institute for Bioengineering and Biosciences, Instituto Superior Técnico, Universidade de Lisboa, Av. Rovisco Pais 1, 1049-001 Lisboa, Portugal, [b]Wake Forest Institute for Regenerative Medicine, 391 Technology Way NE Winston-Salem, NC 27101, USA, [c]Laboratory for Tissue Engineering and Stem Cell Therapy, Department of Urology, University Hospital Zurich, Zurich, Switzerland, [d]Serviço Urologia, Hospital Garcia de Orta, Av. Torrado da Silva 2801-951, Almada, Portugal, [e]Faculdade de Medicina Veterinária, The Interdisciplinary Centre of Research in Animal Health (CIISA), Universidade de Lisboa, Avenida Universidade Técnica, 1300-477 Lisboa, Portugal, [f]IIS Aragón, CIBERehd, Zaragoza, Spain and [g]Instituto Aragonés de Ciencias de la Salud, Centro de Investigación Biomédica de Aragón, Av. San Juan Bosco 13, 50009 Zaragoza, Spain

Corresponding Author:

Pedro M. Baptista

Instituto Investigación Sanitaria de Aragón (IIS Aragón)

Centro de Investigación Biomédica de Aragón

Avenida San Juan Bosco, 13

50009 Zaragoza, Spain

E-mail: pbaptista.iacs@aragon.es; pmbaptista1976@gmail.com

Supplementary Figures

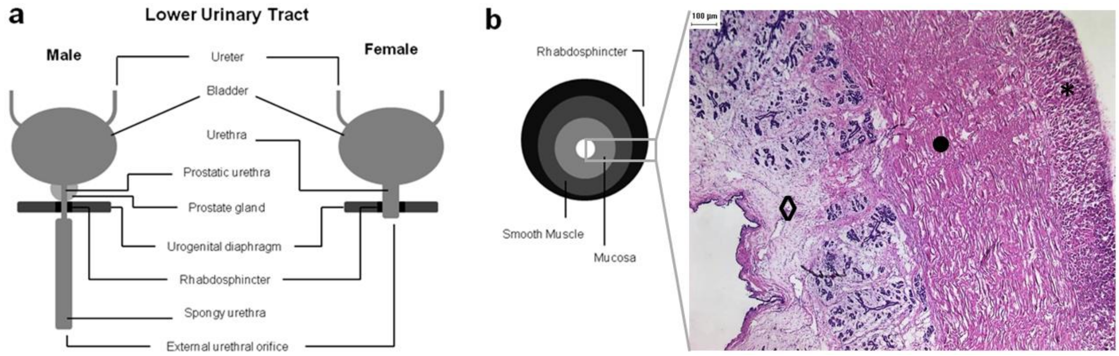

Supplementary Figure S1. Urethra anatomy. (**a**) Schematic representation of the anatomical structure of male and female urethra (**b**) and cross-sectional organization of muscle (●, smooth and *, skeletal or RBS) and mucosal (◊) tissues in the urethra. Scale bar: 100µm. Image obtained through H&E staining.

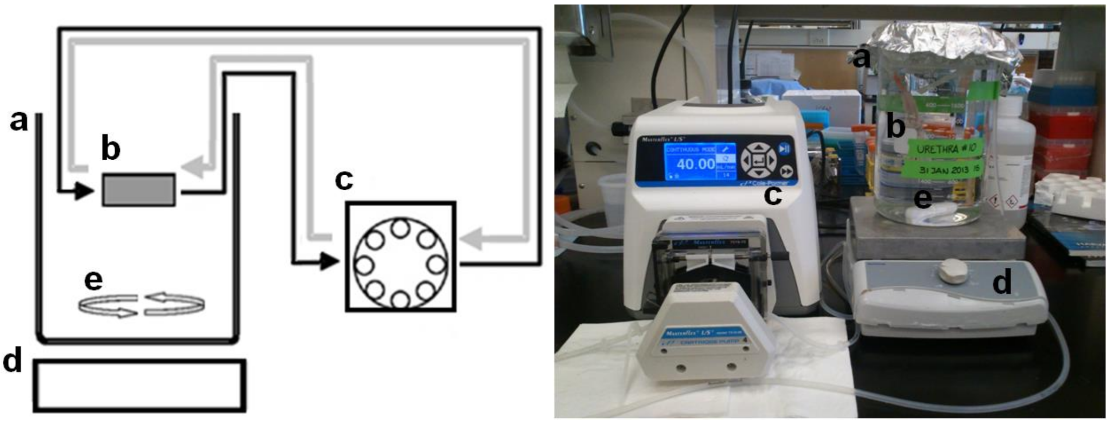

Supplementary Figure S2. Decellularization apparatus based on mechanochemical action. Detergent solution (or distilled water) was placed inside the (**a**) vessel, where the (**b**) urethra was submerged. The solution was pumped by a (**c**) peristaltic pump and recircularized at a rate of 40 mL/min. Through a (**d**) magnetic stirrer plate the solution was (**e**) agitated at 60rpm. The process was repeated for 5 days. By the end of the first 48h, the direction of perfusion was changed (from the black arrows to the grey arrows) to allow a more efficient decellularization.

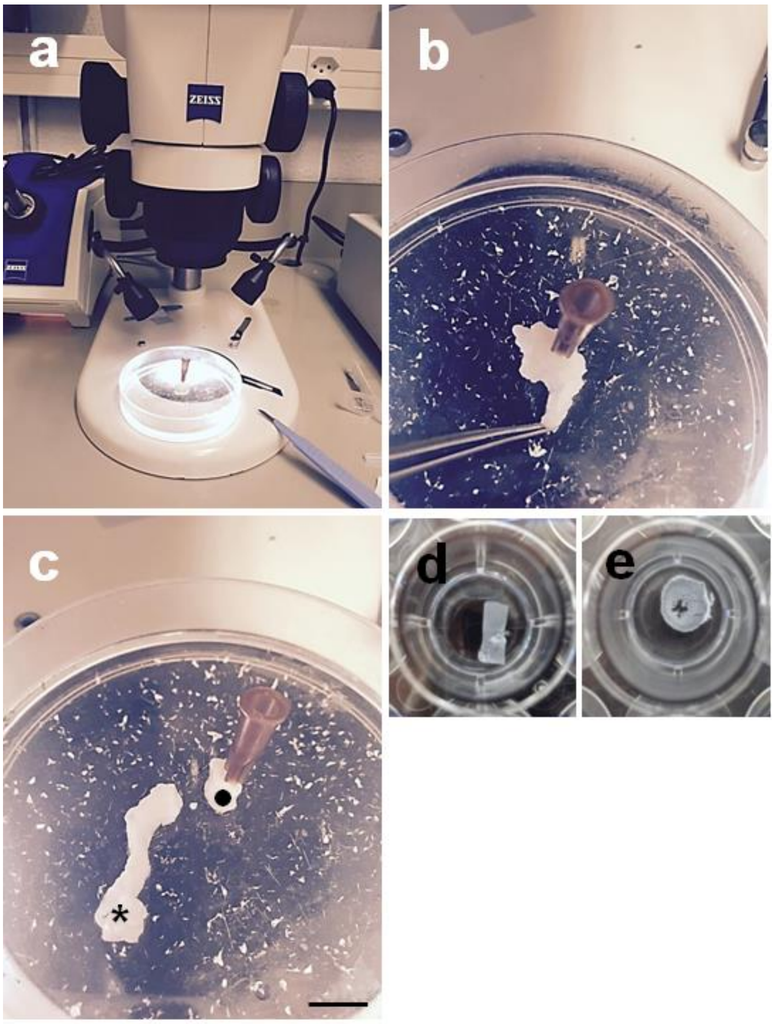

Supplementary Figure S3. Microdissection of the urethra bioscaffolds to prepare sections with different muscle ECMs. (**a**) Microdissection was performed under a stereo microscope and the bioscaffold was pinned on a gelatin coated plate. (**b-c**) RBS (*) was detached from the smooth muscle layer (●) and (**d**) skECM and (**e**) smECM sections, respectively were produced. Scale bar: 1 cm.

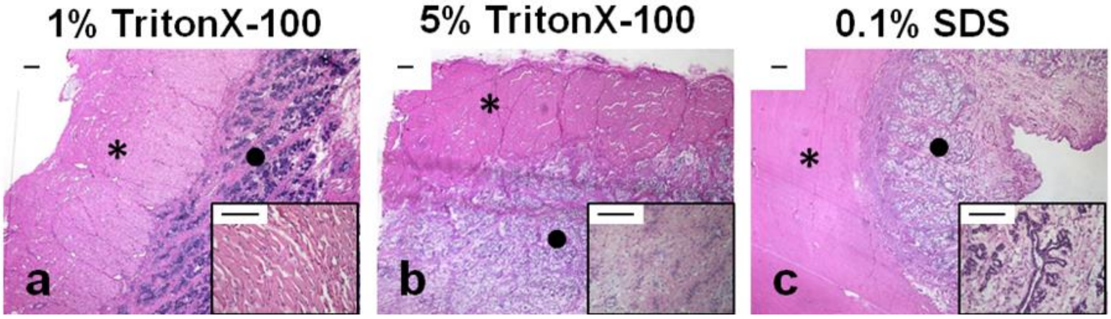

Supplementary Figure S4. H&E staining of urethras exposed to decellularization. (**a**) 1% Triton X-100, (**b**) 5% Triton X-100 and (**c**) 0.1% SDS did not decellularize the urethras. Images are from 5 µm thick sections (*, muscle and ●, mucosa). Scale bar: 100 µm.

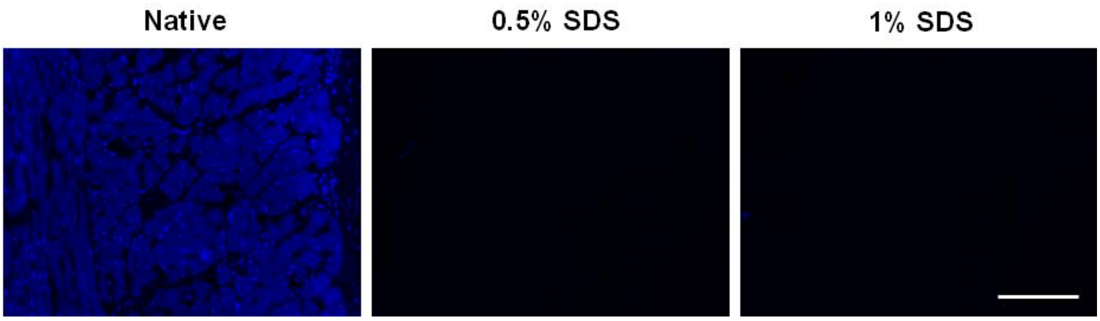

Supplementary Figure S5. DAPI staining of decellularized urethra bioscaffolds. Images are centred on the RBS layer. Images are from 5 µm thick sections. Scale bar: 100 µm.

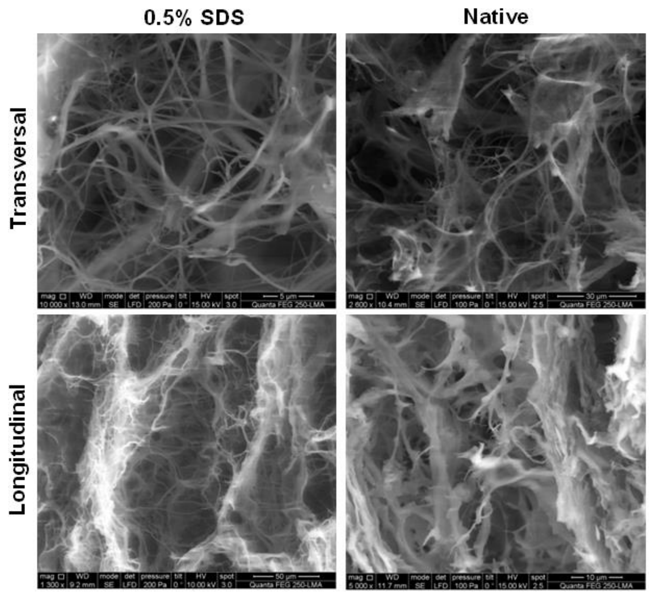

Supplementary Figure S6. SEM of decellularized urethra bioscaffolds. Longitudinal and transversal cuts of native urethra and 0.5% decellularized bioscaffold.

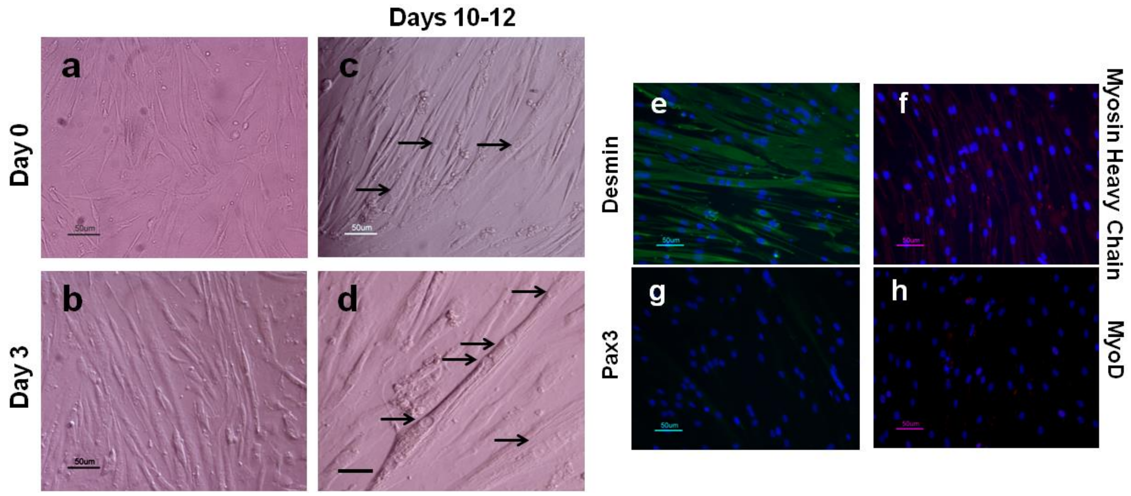

Supplementary Figure S7. MPCs (ZenBio®) fiber formation *in vitro*. Cells changed their morphology after exposure to the differentiation medium at (**a**) days 0 and (**b**) 3. Between (**c-d**)

days 10 and 12 cells fused and produce multinucleated fibers (black arrows). Fibers expressed (**e**) desmin and (**f**) myosin heavy chain while showing no (**g**) Pax3 and (**h**) MyoD. Nuclei were stained with DAPI (in blue). Scale bar: 50 µm.

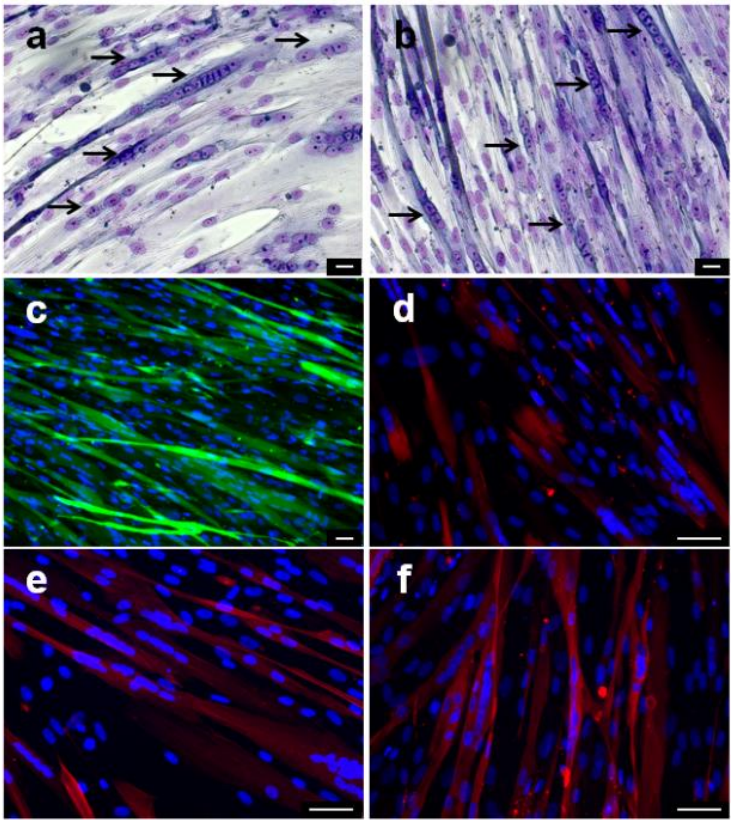

Supplementary Figure S8. MPCs (isolated at USZ) *in vitro* fiber formation. After (**a-b**) 14 days in culture, MPCs fused and produced multinucleated fibers (black arrows) while demonstrating (**c**) viability through calcein AM staining and the expression of (**d**) desmin, (**e**) α-actinin and (**f**) MyHC (all in red) through immunofluorescence staining. Nuclei were stained with DAPI (in blue). Scale bar: 50 µm.

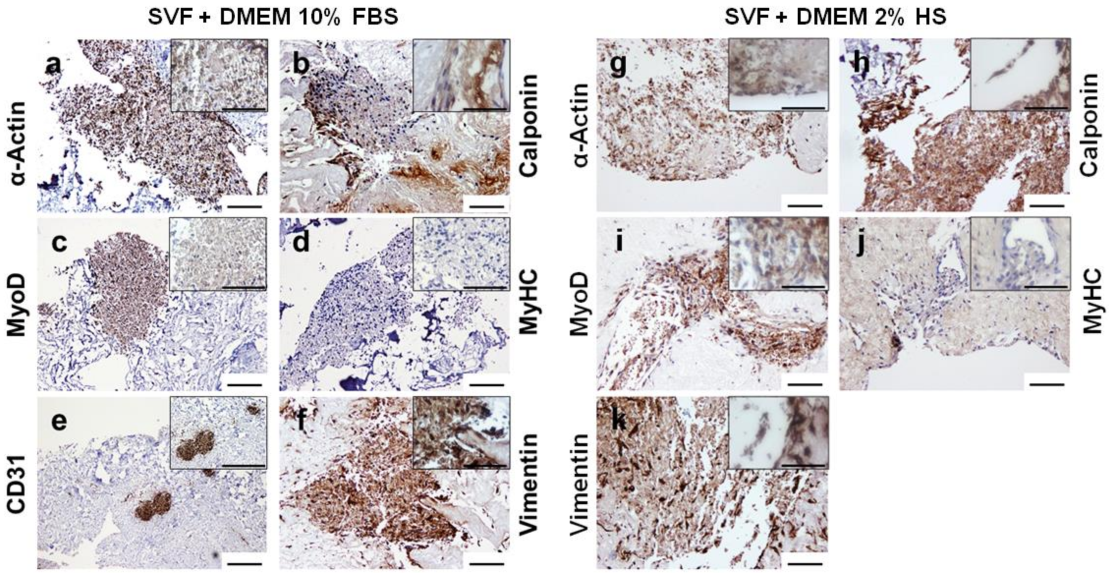

Supplementary Figure S9. Recellularization of urethra bioscaffolds with SVF populations. Cultures were performed using (**a-f**) DMEM + 10%FBS or (**g-k**) DMEM + 2% HS. Presence of (**a** and **g**) α-actin, (**b** and **h**) calponin, (**c** and **i**) MyoD, (**d** and **j**) MyHC, (**e**) CD31 and (**f** and **k**) vimentin was assessed through immunohistochemistry. Scale bar: 100 µm.

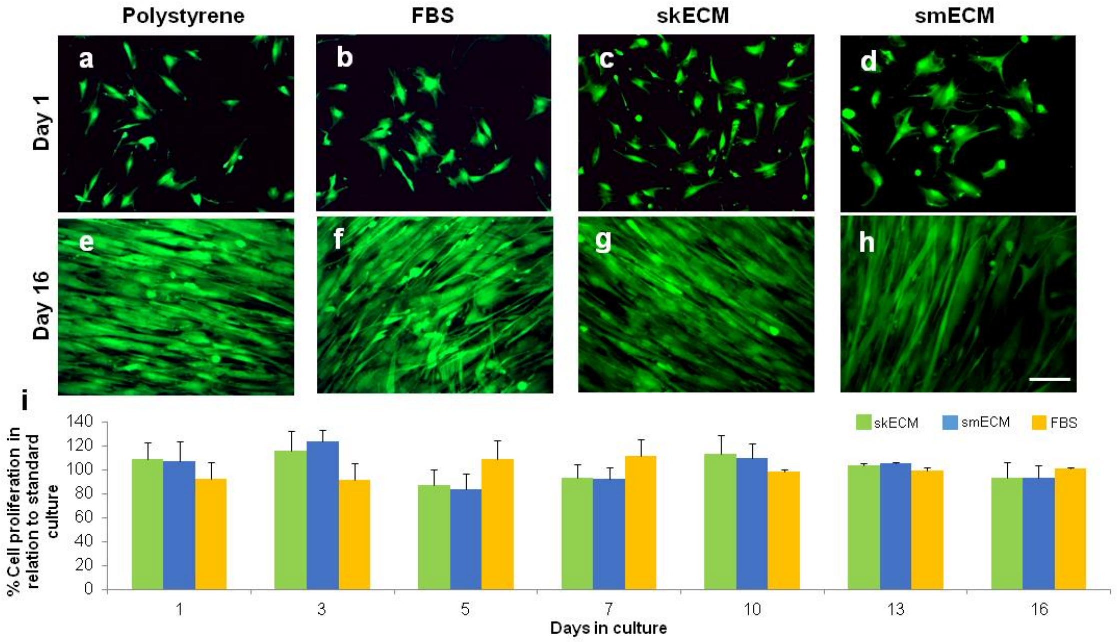

Supplementary Figure S10. Cell viability and proliferation on urethra ECM coatings. (**a-h**) Cell viability was assessed using Calcein AM staining. Scale bar: 150 µm. (**i**) Cell proliferation was assessed using WST-1 assay (Roche) at 450 nm in relation to MPCs grown in non-coated-polystyrene: skECM (green), smECM (blue) and FBS (yellow) coatings. Results as mean ± SEM (n=3), $p > 0.05$ for all time points, ANOVA.

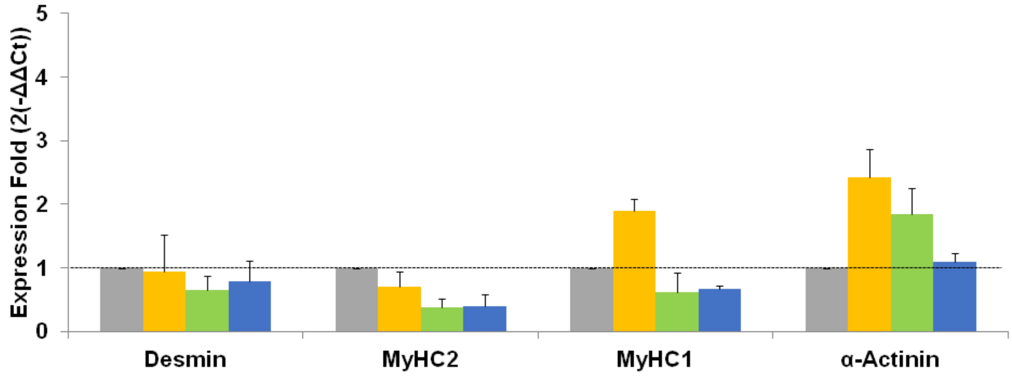

Supplementary Figure S11. Expression of skeletal muscle-related genes in MPCs on urethra ECM coatings. The expression of desmin, MyHC and α-actinin genes was quantified through RT-qPCR on MPCs after 14 days of differentiation in non-coated polystyrene (grey), FBS (yellow), skECM (green) and smECM (blue). Dashed line marks the expression of the gene in the reference condition, non-coated polystyrene (grey). Results as mean ± SEM (n=3), $p > 0.05$ for all conditions, ANOVA.

Supplementary Table S1. Data for the estimation of the pressure (P) to which the urethra is subjected with the perfusion decellularization method.

14 Gauge Internal Diameter	0.0016 m
14 Gauge Length	0.0450 m
Area	2.01×10^{-6} m^2
ρ Water	1000 Kg/m^3
ρ Triton X-100	1070 Kg/m^3
ρ SDS	1030 Kg/m^3
Flow Rate (Q)	6.67x10-7 m^3/s
Velocity (V)	0.33 m/s

Supplementary Table S2. Macroscopic assessment of the outcome of the decellularization process using each detergent solution tested (+ for positive decellularization, organ become white/transparent; – for negative decellularization, organ did not become white/transparent).

Decellularization Solution	Results
1% Triton X-100 + 0.1% NH_4OH	-
5% Triton X-100 + 0.5% NH_4OH	-
0.1% SDS	-
0.5% SDS	+
1% SDS	+

Supplementary Video S1. MPCs fiber formation *in vitro*. After 12 days of induction MPCs started to contract. Scale bar: 100 µm. Time frame: 9 seconds.

Supplementary Methods

Decellularization of Urethra Tissue – DAPI Staining

Five µm thick sections of native (n=4) and decellularized (n=5, 0.5% SDS and n=2, 1% SDS) urethras processed for DAPI staining. DAPI (1:100, Sigma®) was incubated for 1 h at room temperature. Slides were mounted with ProLong® Gold Antifade (Invitrogen™). Images were captured on a Leica Microsystems DM4000B upright microscope (Leica Microsystems Inc., Wetzlar, Germany) with Image-Pro® Express software (Media Cybernetics Inc., Rockville, MD, USA).

Isolation, Expansion and Differentiation of Cells for Recellularization and Seeding

Muscle Progenitor Cells (MPCs)

MPCs were purchased (Zenbio®) or isolated from muscle tissue obtained from healthy donors after informed consent at USZ in Switzerland[25]. MPCs from Zenbio® were plated at 5000 cells/cm^2 and expanded using SkMGM (ZenBio®) for at least two cell passages. MPCs isolated at USZ were plated at 5000 cells/cm^2 and expanded for at least one cell passage using growth medium DMEM/F12 (Gibco®) supplemented with 18% FBS (Gibco®), 10 ng/ml hEGF (Sigma©), 1 ng/ml hbFGF (Sigma©), 10 µg/ml human insulin (Sigma©) and 0.4 µg/ml dexamethasone (Sigma©)).

Stromal Vascular Fractions (SVF)

SVF were isolated from discarded liposuctions of healthy donors after informed consent at "Clínica de Todos-Os-Santos" in Lisbon, Portugal using a standard enzymatic protocol[26]. SVF populations were immediately used for recellularization.

Bone Marrow Mesenchymal/Stromal Stem Cells (BM MSCs) and Fibroblasts

BM MSC were isolated from bone marrow aspirates obtained from healthy donors at Instituto Português de Oncologia Francisco Gentil in Lisbon, Portugal (IPOFG-L), after informed consent from an approved protocol by the Ethical Review Board of IPOFG-L[27]. BM MSC and mouse

L929 fibroblasts (Leibniz-Institute DSMZ) were plated at 3000 cells/cm^2 and expanded using DMEM + 10% FBS for at least one cell passage. All cultures were maintained at 37ºC and 5% CO_2 with medium changes every 3-4 days.

Microdissection of Urethra Bioscaffolds

The RBS layer of whole decellularized urethras (n=4) was isolated from the remaining bioscaffold through microdissection (Supplementary Figure S3). Transversal fragments of the area containing the RBS layer were cut and pinned on gelatin-coated plates. Microdissection was carried out under stereo microscope (Supplementary Figure S3a, Zeiss Stemi 2000-c, Carl Zeiss©) and using a Zeiss KL1500 LCD cold light source (Carl Zeiss©). A small incision was made on the most external layer of the bioscaffold (Supplementary Figure S3b) using a disposable scalpel. The RBS was detached from the remaining bioscaffold using the blunt end of the scalpel (Supplementary Figure S3c), creating two bioscaffolds – skECM (skeletal muscle ECM, Supplementary Figure S3d) and smECM (smooth muscle ECM, Supplementary Figure S3e).

MPCs Fiber Formation

Fiber formation was induced using DMEM/F12 + 10% FBS (Gibco®) (DMEM + 2% HS (Gibco®) for the MPCs from Zenbio®) for 14 days at 37ºC and 5% CO_2 with medium changes every 3 days. Fiber formation was confirmed through immunofluorescence staining using anti-desmin (1:50, Sigma©), anti-Pax3 (1:100, Santa Cruz Biotech.), anti-MyoD (1:100, BD Biosciences), anti-α-actinin (1:500, Sigma©) and anti-MyHC (1:1, DSHB, University of Iowa, IO, USA). Secondary antibodies FITC-conjugated goat anti-mouse (Abcam®, Cambridge, UK), FITC-conjugated donkey anti-goat, Alexa-546-conjugated goat anti-mouse, Alexa-546-conjugated goat anti-rabbit (all 1:500, all from Invitrogen™) and Cy3-conjugated sheep anti-mouse (1:1000, Sigma©) were used. Cell nuclei were counterstained using DAPI (1:10000, Sigma®). For Pax3 staining all solutions were prepared using FBS instead of NGS. Images were acquired using Leica DM6000 B fluorescence microscope and the LAS AF software both from Leica Microsystems Inc.

Cell Viability Analysis

Cultures were washed with PBS and incubated in culture medium containing Calcein AM (1:1000, Sigma©) for 30 min. Images were acquired using Leica DM6000 B fluorescence microscope and the LAS AF software both from Leica Microsystems Inc.

RT-qPCR

Total RNA was isolated with SV Total RNA Isolation System Kit (Promega) according to manufacturer's instructions. RNA concentration was determined through spectrophotometry at 260 nm using NanoDrop Lite (Thermo-Scientific™). RNA was reverse transcribed with random primers using the High Capacity cDNA Reverse Transcription Kit (Applied Biosystems™) and the T3000 Thermocycler (Biometra). Pre-designed primers for human desmin, MyHC1, MyHC2 and α-actinin were purchased from Life Technologies™. The analysis was normalized to the endogeneous gene S18. Real Time quantitative Polymerase Chain Reaction (RT-qPCR) was performed using RotorGene RG3000 and data was acquired using RotorGene software (both from CR Corbett Research). RT-qPCR was performed based on TaqMan assay, TaqMan Fast Advanced Master Mix (Applied Biosystems™). Detection was made with Fluorophor FAM and wavelength of 495 nm. The expression fold of each gene was calculated using Supplementary Equation (S1), where ΔCt is the C_{target} - $Ct_{endogenous}$ (Ct, threshold cycle). The cultures using non-coated polystyrene were used as the reference condition for all calculations.

$$Expression\ Fold = 2^{(-(\Delta Ct_{target} - \Delta Ct_{reference}))} \qquad \text{Supplementary Equation (S1)}$$
